# The effect of hyperlipidemia and body fat distribution on subclinical left ventricular function in obesity: a cardiovascular magnetic resonance study

**DOI:** 10.1186/s12933-024-02208-z

**Published:** 2024-04-02

**Authors:** Jing Liu, Jing Li, Chunchao Xia, Wenzhang He, Xue Li, Sumin Shen, Xiaoyue Zhou, Nanwei Tong, Liqing Peng

**Affiliations:** 1 Department of Radiology, West China Hospital, Sichuan University, 37 Guoxue Alley, Chengdu, 610041 China; 2 Department of Endocrinology and Metabolism, Center for Diabetes and Metabolism Research, West China Hospital, Sichuan University, 37 Guoxue Alley, Chengdu, 610041 China; 3 MR Collaboration, Siemens Healthineers Ltd., Shanghai, 200126 China

**Keywords:** Ventricular dysfunction, Obesity, Body fat distribution, Hyperlipidemia, Dual-energy X-ray absorptiometer, Strain

## Abstract

**Background:**

Obesity is often associated with multiple comorbidities. However, whether obese subjects with hyperlipidemia in the absence of other complications have worse cardiac indices than metabolically healthy obese subjects is unclear. Therefore, we aimed to determine the effect of hyperlipidemia on subclinical left ventricular (LV) function in obesity and to evaluate the association of cardiac parameters with body fat distribution.

**Materials and methods:**

Ninety-two adults were recruited and divided into 3 groups: obesity with hyperlipidemia (n = 24, 14 males), obesity without hyperlipidemia (n = 25, 13 males), and c ntrols (n = 43, 25 males). LV strain parameters (peak strain (PS), peak diastolic strain rate (PDSR), peak systolic strain rate) derived from cardiovascular magnetic resonance tissue tracking were measured and compared. Dual-energy X-ray absorptiometer was used to measure body fat distribution. Correlations of hyperlipidemia and body fat distribution with LV strain were assessed by multivariable linear regression.

**Results:**

Obese individuals with preserved LV ejection fraction showed lower global LV longitudinal, circumferential, and radial PS and longitudinal and circumferential PDSR than controls (all *P* < 0.05). Among obese patients, those with hyperlipidemia had lower longitudinal PS and PDSR and circumferential PDSR than those without hyperlipidemia (− 12.8 ± 2.9% vs. − 14.2 ± 2.7%, 0.8 ± 0.1 s^−1^ vs. 0.9 ± 0.3 s^−1^, 1.2 ± 0.2 s^−1^ vs. 1.4 ± 0.2 s^−1^; all *P* < 0.05). Multivariable linear regression demonstrated that hyperlipidemia was independently associated with circumferential PDSR (β = − 0.477, * P* < 0.05) in obesity after controlling for growth differences, other cardiovascular risk factors, and central fat distribution. In addition, android fat had an independently negative relationship with longitudinal and radial PS (β = − 0.486 and β = − 0.408, respectively; all* P* < 0.05); and visceral fat was negatively associated with longitudinal PDSR (β = − 0.563, *P* < 0.05). Differently, gynoid fat was positively correlated with circumferential PS and PDSR and radial PDSR (β = 0.490, β = 0.481, and β = 0.413, respectively; all* P* < 0.05).

**Conclusion:**

Hyperlipidemia is independently associated with subclinical LV diastolic dysfunction in obesity. Central fat distribution (android and visceral fat) has a negative association, while peripheral fat distribution (gynoid fat) has a positive association on subclinical LV function. These results suggest that appropriate management of hyperlipidemia may be beneficial for obese patients, and that the differentiation of fat distribution in different regions may facilitate the precise management of obese patients.

*Clinical trials registration* Effect of lifestyle intervention on metabolism of obese patients based on smart phone software (ChiCTR1900026476).

**Supplementary Information:**

The online version contains supplementary material available at 10.1186/s12933-024-02208-z.

## Introduction

The global prevalence of overweight and obesity has markedly increased in recent decades, and obesity has become an epidemic in China, with an estimated increase in adults of 0.3% per year over fourteen years (2000–2014) [1]. Obesity often exists alongside comorbidities, such as hypertension, diabetes, and coronary heart disease, increasing cardiovascular disease events and mortality [2, 3]. However, it is unclear whether these patients who were only found to be hyperlipidemic and lacked other complications need aggressive intervention, such as lipid-lowering and cardiovascular protection. One of our study aims was to determine whether obese subjects with hyperlipidemia free from other complications have worse cardiac parameters than metabolically healthy obese subjects.

Compared with echocardiography, cardiovascular magnetic resonance (CMR) with a large field of view, better image quality, and three-dimensional (3D) cine images of the heart, aids in assessing ventricular geometry and function with high accuracy and reproducibility[4, 5]. Moreover, due to signal interference caused by excessive adiposity, the application of echocardiography in obese individuals is limited. Thus, CMR may be a preferred method to evaluate cardiac structure and function in obese patients. Previous studies have shown that obesity is a strong risk factor for heart failure, particularly for heart failure with preserved ejection fraction (EF) [6–8]. EF was insensitive to the myocardial changes related to obesity [9]. CMR tissue tracking is widely used to detect early myocardial dysfunction with preserved LVEF, due to its high sensitivity in measuring global and regional cardiac deformation in directions through the tracking of myocardial motion [10], In addition, CMR tissue tracking has good intra- and inter-observer reproducibility across different postprocessing software vendors [11].

Body mass index (BMI), as an index of general obesity, has been associated with increased risks of cardiovascular morbidity and mortality [12]. However, previous studies have found that regions of fat deposition have various effects on the heart [13, 14]. Dual X-ray absorptiometry (DXA) is widely used for assess body fat distribution. Several studies have reported the associations of LV remodeling or peak circumferential strain with DXA-derived fat distribution parameters, such as total body fat, lean mass, lower body fat, and visceral or subcutaneous fat [15–17]. A recent study revealed the different effects of central and peripheral fat distributions on right ventricular function [18]. In this study, DXA was applied to further subdivided regional fat distribution, including fat mass in android, gynoid, trunk, upper and lower extremity, and visceral regions, and to explore the relationship between fat in these regions and LV strain in different directions (radial, circumferential, and longitudinal).

This study aimed to determine the association between hyperlipidemia and subclinical LV function based on CMR tissue tracking in obesity, and to evaluate relationship between DXA-associated body fat distribution and LV function parameters.

## Methods and materials

### Study population

The study complied with the Declaration of Helsinki and was approved by the Institutional Review Board of West China Hospital of Sichuan University. Written informed consent was obtained from all study participants before undergoing CMR examinations.

This was an exploratory study. We recruited 49 obese subjects defined by a BMI ≥ 27.5 kg/m^2^ (range, 27.5–34.9 kg/m^2^) and 43 healthy volunteers (18.5 ≤ BMI ≤ 23 kg/m^2^) [19, 20] between 18 and 60 years old from September 2019 to June 2022. Subjects were excluded if they had any of the following conditions: hypertension (systolic blood pressure [SBP] ≥ 140 mmHg and diastolic blood pressure [DBP] ≥ 90 mmHg) and diabetes measured by oral glucose tolerance; history of lipid-lowering, hypoglycemic or antihypertensive drugs; history of cardiovascular diseases or history of any cardiovascular procedures; major systemic diseases that could affect the myocardium, such as connective tissue diseases and endocrine diseases; respiratory diseases that could affect the heart, such as chronic obstructive pulmonary emphysema and obstructive sleep apnea; infection, fever, and renal failure; or any contraindications to CMR imaging. According to the criteria for Asian or Chinese population, BMI was categorized into three groups: healthy weight (18.5–23.0 kg/m^2^), overweight (23.0–27.5 kg/m^2^), and obese (≥ 27.5 kg/m^2^). Mild, moderate, and severe obesity were defined as 27.5 kg/m^2^ ≤ BMI < 32.5 kg/m^2^, 32.5 kg/m^2^ ≤ BMI < 37.5 kg/m^2^, and BMI ≥ 37.5 kg/m^2^ [19, 20]. Obese patients were divided into two subgroups, one with hyperlipidemia (obese (hyperlipidemia +) group: n = 24; median age 31 years; 14 males) and the other without hyperlipidemia (obese (hyperlipidemia-) group: n = 25; median age 29 years; 13 males), also labelled as metabolically healthy obesity. Age- and sex-matched volunteers were included in a healthy control group (n = 43; median age 29 years; 25 males).

### Baseline data collection

Baseline data of the participants were collected, including medical history, weight, height, heart rate, and blood pressure (BP). Fasting blood glucose (FBG), fasting insulin (FINS), and serum lipid profiles, including triglycerides, total cholesterol, high-density lipoprotein (HDL), low-density lipoprotein (LDL), and very-low-density lipoprotein were also measured. Mean arterial pressure (MAP; mmHg) was calculated as follows: MAP = (SBP + 2DBP)/3, respectively.

Homeostasis model assessment of insulin resistance (HOMA-IR) was calculated as follows: HOMA-IR = [FBG (mmol/L) × FINS (mU/L)]/22.5.

Dyslipidemia was defined when one of the following criteria was met: (1) triglycerides > 1.7 mmol/L; (2) total cholesterol > 5.7 mmol/L, (3) LDL > 4.3 mmol/L, (4) HDL < 0.8 mmol/L [21].

### Assessment of obesity

**Fig. 1:**
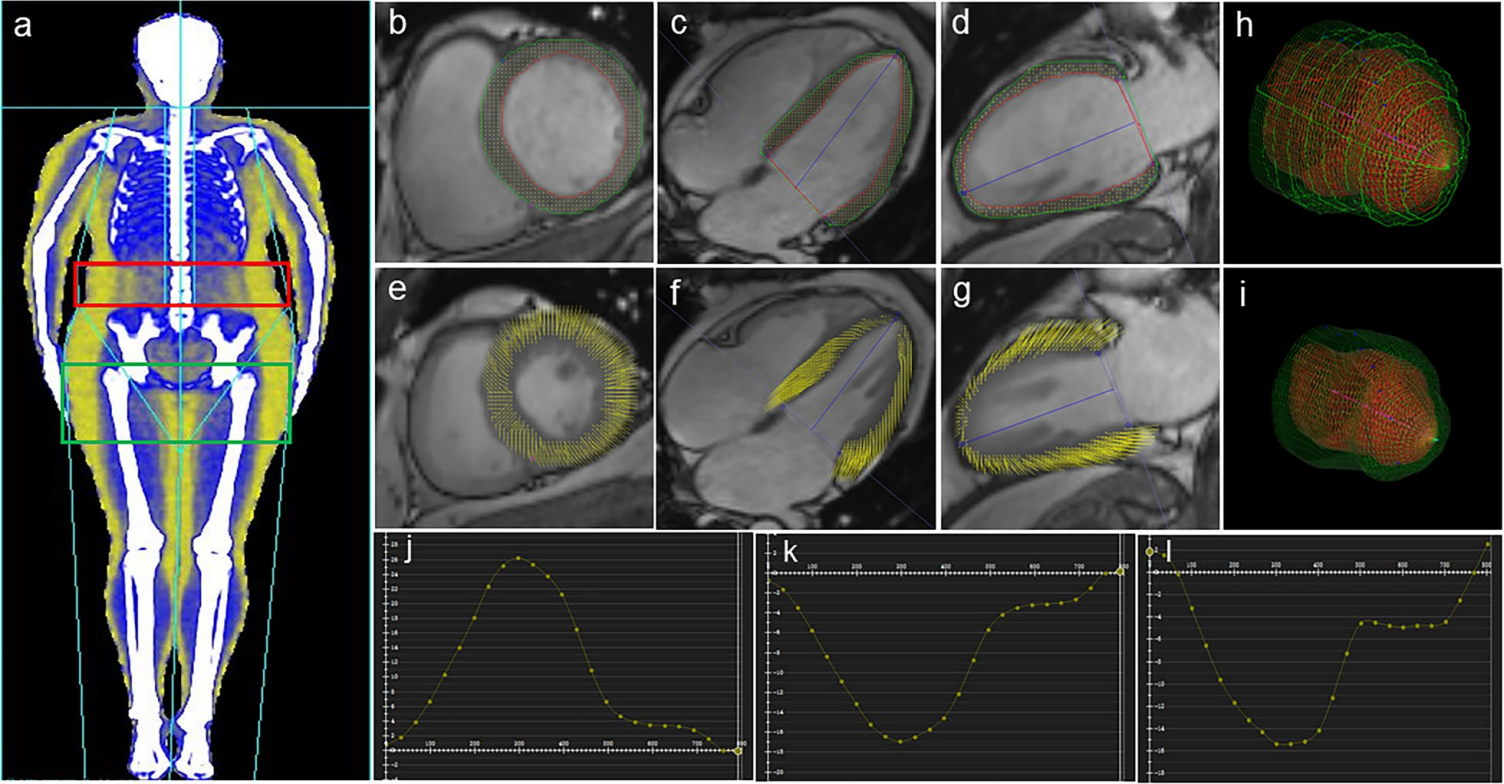
Body fat distribution with dual X-ray absorptiometry and LV strain using cardiac magnetic resonance imaging. **a** Segmentation diagram of the whole-body fat distribution. The red and green boxes represent the android fat and gynoid fat regions, respectively. **b**–**d** LV contours are delineated on a short-axis, two-chamber, and four-chamber views at the end-diastolic phase. **e**–**g** Using tissue tracking, LV myocardial points motion from the end-diastolic phase to the end-systolic phase. **h** and **i** 3D models of the LV at the end-diastolic and end-systolic phases. **J**–**l** LV strain curves, radial, circumferential, and longitudinal strains in order

BMI (kg/m^2^) was calculated as weight (kg) divided by height squared (m^2^). Waist circumference (WC) and hip circumference (HC) were measured. Body fat distribution was measured using DXA (Lunar iDXA, GE Medical Systems Lunar) and as shown in Fig. 1a. Percentages of fat mass in android, gynoid, trunk, peripheral, upper extremity, lower extremity, and visceral regions reflected fat deposition in the corresponding regions, relative to the total fat mass. In addition, Percentage of fat mass in peripheral region was calculated as the sum of the upper and lower extremity fat mass percentages. Percentages of fat mass in the android, trunk, and visceral regions were indices predictive of central obesity; while percentages of fat mass in gynoid, peripheral, upper extremity, and lower extremity regions were indices predictive of peripheral obesity.

### CMR protocol

CMR examinations were performed using a 3 Tesla whole-body scanner (MAGNETOM Skyra, Siemens Healthcare) with an 18-channel phased-array coil. With a standard electrocardiograph-triggering device, data were acquired during the end-expiratory breath-hold period. Contiguous cine images in the short-axis view from the base to the apex and the two- and four-chamber cine images in the long-axis were obtained by a balanced steady-state free precession (bSSFP) sequence. The scan parameters were as follows: repetition time/echo time = 3.3/1.22 ms, flip angle = 41°, slice thickness = 8 mm, and a temporal resolution = 39.34 ms, field of view = 360 mm × 320 mm; and matrix size = 208 × 166.

### CMR image analysis

All MRI data were imported to commercially available software (CVI 42 version 5.11.3, Circle Cardiovascular Imaging Inc). Two radiologists with more than three years of CMR experience completed the image analysis and were blinded to the subject status (control vs. obesity).

#### Global LV geometry and function

The endocardial and epicardial contours of the LV myocardium were manually traced during the end-diastolic and end-systolic phases on the short-axis cine images. The global geometry and function parameters, namely end-diastolic volume (EDV), end-systolic volume (ESV), EF, and LV mass at end-diastole, were automatically computed. LV mass was indexed to the height^2.7^ [LV mass index (LVMI); g/m^2.7^] (13). Concentricity was calculated as the ratio of LV mass to LVEDV (g/mL). In addition, mean LV regional values for sixteen myocardial segment thicknesses (excluding the apex) were also automatically computed [American Heart Association standard seventeen-segment model (14)]. Finally, the LV maximum myocardial thickness (LVMMT) was calculated.

#### LV strain

The long-axis four-chamber, two-chamber, and short-axis cine slices were transferred to the 3D tissue tracking module for LV myocardial strain analysis. The endocardial and epicardial contours were manually delineated per slice during the end-diastolic phase. For different directions of myocardial deformation, LV global myocardial strain parameters, including the radial, circumferential, and longitudinal peak strains (PS), peak systolic strain rates (PSSR), and peak diastolic strain rates (PDSR) can be calculated. (Fig. 1b–l).

#### Epicardial adipose tissue (EAT) quantification

EAT was defined as a high-signal intensity region between the myo-epicardium and pericardium. The measurement method has been described elsewhere [22].

### Reproducibility

Intra- and inter-observer variabilities for LV myocardial strain indices were analyzed in 40 random subjects, including twenty obese patients and twenty healthy controls. To determine intra-observer variability, one radiologist measured the same image over a one-month interval. To evaluate the inter-observer variability, the second radiologist, who was blinded to the first observer’s results, re-analyzed the measurements.

### Statistical analysis

All statistical analyses were performed using SPSS software (version 23, IBM, Armonk, Armonk, New York, USA). Normally distributed continuous data were expressed as the means ± standard deviation, while non-normally distributed data were expressed as the median (25–75% interquartile range). The differences between all obesity and healthy controls were compared using Student’s t-test or the Mann–Whitney U test as appropriate. Categorical data were presented as numbers (percentages) and compared by the chi-square or Fisher’s exact test. In addition, Kruskal–Wallis test (non-normally distributed data or unequal variances) or the one-way analysis of variance (ANOVA) (normally distributed data and equal variances) was applied to compare continuous data among healthy control, obesity (hyperlipidemia-), and obesity (hyperlipidemia +) groups. Pearson’s and Spearman’s correlation coefficient was used to determine correlations between body fat distribution, cardiovascular risk factors, and LV function parameters in whole study population. A stepwise multivariable linear regression was used to observe the correlation between hyperlipemia and LV strain in obesity. The metabolic variables, including FBG, FINS, HOMA-IR, and MAP as confounding factors were added. FINS was excluded because FINS was collinearity with HOMA-IR. In addition, central fat distribution (visceral fat or android fat or trunk fat) as a confounder was added due to an established connection between central obesity and dyslipidemia. Furthermore, the stepwise multivariable linear regression was also used to explore the relationship between body fat distribution and LV strain parameters in whole study population. Body fat distribution, including android fat, gynoid fat, trunk fat, peripheral fat, visceral fat, WC, waist-to-hip ratio, and EAT were entered in univariable analyses. Growth differences data (age, sex, and height) were added in all multivariable analyses. Variables with a *P* < 0.1 in the univariable analyses were then included in a stepwise multivariable analysis. Finally, the intraclass correlation coefficient (ICC) was used to evaluate both inter-and intra-observer variabilities. A *P* < 0.05 indicated statistical significance.

## Results

### Baseline characteristics among the four groups

This was a study of 49 obese adults (24 patients with hyperlipidemia, 25 patients without hyperlipidemia) and 43 healthy controls. Among obese patients, 42 were mild obesity (85.7%) and 7 were moderate obesity (14.3%). Their baseline characteristics are shown in Table 1. Median age, gender, height, and heart rate were compared, and the differences between groups were not statistically significant (*P* > 0.05). Although BP was within the normal range in all groups, obese subjects had higher SBP, DBP, and MAT compared with healthy controls (all *P* < 0.05), and these parameters were similar in two obese subgroups.

**Table 1:** Baseline characteristics and body fat distribution among the four groups

Parameters	Controls (n = 43)	All obesity (n = 49)	Obesity	
			Hyperlipidemia-(n = 25)	Hyperlipidemia + (n = 24)
Baseline characteristics				
Male, %	25 (58.1)	27 (55.1)	13 (52)	14 (58.3)
Age (years)	29.0 (24.0, 34.0)	30.0 (26.0, 37.5)	29.0 (25.0, 37.5)	31.0 (26.5, 38)
Height (cm)	166.6 ± 8.3	167.6 ± 9.4	167.1 ± 8.5	168 ± 10
Body weight (kg)	53.7 (50.0, 62.0)	84.0 (75.0, 91.0)*	81.5 (71.3, 91.0)*	85.0 (78.1, 93.2)*
BMI (kg/m^2^)	19.9 (18.8, 20.9)	28.4 (29.4, 31.1)*	28.6 (28.0, 30.4) *	30.0 (29.4, 31.2)*§
Heart rate (bpm)	74.2 ± 8.0	73.7 ± 9.6	73.7 ± 9.5	73.8 ± 9.9
SBP (mmHg)	109 ± 11	124 ± 10*	123 ± 11*	124.4 ± 9.0*
DBP (mmHg)	71.6 ± 8.8	78.9 ± 6.5*	77.1 ± 6.5*	80.8 ± 6*
MAP (mmHg)	83.9 ± 9.2	93.8 ± 6.6*	92.2 ± 6.7*	95.4 ± 6.3*
FBG (mmol/L)	4.8 ± 0.3	5.4 ± 0.6*	5.2 ± 0.7*	5.5 ± 0.5*§
Total cholesterol (mmol/L)	4.0 ± 0.7	4.9 ± 1.1*	4.3 ± 0.8	5.4 ± 1*§
Triglycerides (mmol/L)	0.5 (0.4, 0.7)	1.6 (1.0, 2.7)*	1.0 (0.7, 1.4)	2.6 (1.9, 3.5) *§
HDL (mmol/L)	1.6 ± 0.4	1.3 ± 0.3*	1.3 ± 0.2*	1.2 ± 0.3*
LDL (mmol/L)	2.1 ± 0.5	2.7 ± 0.8*	2.4 ± 0.7*	2.9 ± 0.9*§
VLDL (mmol/L)	0.2 (0.2, 0.3)	0.7 (0.5, 1.2)*	0.5 (0.3, 0.6)*	1.2 (0.9, 1.6)*§
FINS (mmol/L)	5.9 (3.7, 7.7)	13.8 (10.9, 20.4)*	11.9 (9.7, 15.0)*	15.8 (12.3, 27.4)*§
HOMA-IR	1.2 (0.8, 1.6)	3.4 (2.7, 4.8)*	3.0 (2.3, 3.5)*	4.2 (3.6, 7.0)*§
Adiposity measurement				
EAT (cm^3^)	17.9 (14.2, 23.1)	46.2 (37.7, 56.3)*	44.2 (37.6, 57.3)*	47.8 (38.1, 60.5)*
Total fat (kg)	11.5 ± 2.6	29.5 ± 5.1*	28.2 ± 6.5*	30.4 ± 4.5*
Trunk fat (%)	45.5 ± 5	57.7 ± 4.7*	55.6 ± 4.0*	60.0 ± 4.5*§
Peripheral fat (%)	46.5 ± 5.5	38.5 ± 5*	40.4 ± 4.2*	36.4 ± 4.9*§
Upper extremities fat (%)	10.9 ± 1.1	10.5 ± 1.5	11.0 ± 1.3	9.9 ± 1.6*§
Lower extremities fat (%)	35.6 ± 5.1	28 ± 4.2*	29.4 ± 3.9*	26.5 ± 4.1*§
Android fat (%)	5.8 ± 1.2	9.8 ± 1.5*	9.2 ± 1.3*	10.4 ± 1.5*§
Gynoid fat (%)	18.0 ± 2.9	14.9 ± 2.0*	15.4 ± 1.7*	14.4 ± 2.2*
Visceral fat (%)	2.2 (0.9, 3.3)	4.2 (3.3, 5.9)*	3.6 (3.0, 4.8)*	5.3 (3.6, 7.4)*§
Waist circumference (cm)	73.4 ± 5.2	100 ± 11*	99 ± 13*	100.1 ± 8.7*
Hip circumference (cm)	92.6 ± 4.2	107.3 ± 4.1*	107.3 ± 4.3*	107.3 ± 4.1*
Waist-to-hip ratio	0.79 ± 0.05	0.93 ± 0.09*	0.9 ± 0.1*	0.93 ± 0.07*
Waist-to-height ratio	0.44 ± 0.04	0.6 ± 0.06*	0.6 ± 0.07*	0.6 ± 0.04*

Note: *SBP* systolic blood pressure; *DBP* diastolic blood pressure; *MAP* mean arterial pressure; *FBG* fasting blood glucose; *HDL* high-density lipoprotein; *LDL* low-density lipoprotein; *VLDL* very-low-density lipoprotein; *FINS* fasting insulin; *HOMA-IR* homeostasis model assessment of insulin resistance; *EAT* epicardial adipose tissue. *P < 0.05 obese patients versus normal controls; §*P* < 0.05 obese patients with hyperlipidemia + versus obese patients with hyperlipidemia-

For the blood parameters, the obese (hyperlipidemia +) group had higher FBG, FINS, HOMA-IR, triglycerides, total cholesterol, VLDL, and LDL compared to the healthy group and obese (hyperlipidemia-) group (all *P* < 0.05). The obese (hyperlipidemia-) group had higher FBG, FINS, HOMA-IR, VLDL, and LDL compared with the healthy group (all *P* < 0.05). In contrast, all obese groups had lower HDL compared with the healthy group (*P* < 0.05).

In addition, the obese groups had greater conventional fat indices, including WC, HC, waist-to-hip ratio, and waist-to-height ratio compared to the healthy group (all P < 0.05). The obese groups had greater EAT than the healthy control group (P < 0.05). Additionally, the obese groups had greater DXA-related central fat deposition indices, including trunk fat, visceral fat, and android fat compared to the healthy group (all P < 0.05). Among obese patients, those with hyperlipidemia had greater central fat deposition indices than those without hyperlipidemia (all P < 0.05). In contrast, the obese groups had lower DXA-related peripheral fat deposition indices, including gynoid fat, peripheral fat, and lower extremity fat compared with healthy individuals (all P < 0.05). In addition, obese individuals with hyperlipidemia had lower peripheral fat and upper and lower extremity fat than those without hyperlipidemia (all P < 0.05).

### Comparison of CMR findings between the obese subjects and healthy controls

**Fig. 2:**
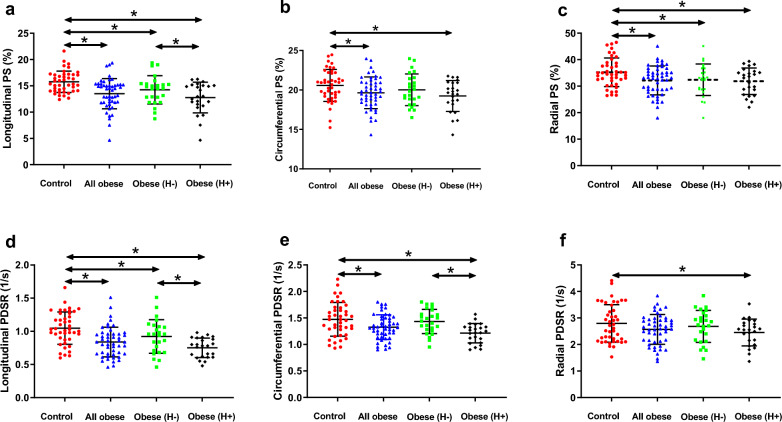
Dot plots comparing the left ventricular strain parameters of patients with obesity and healthy controls. **a**–**c** Global longitudinal, circumferential, and radial PS; **d**–**f** Global longitudinal, circumferential, and radial PDSR. Obese (H-) and obese (H** +**) represent the obese patients without and with hyperlipidemia, respectively. *P < 0.05. *PS* peak strain, *PDSR* peak diastolic strain rate

The LVEFs were within the normal range (LVEF > 50.0%) for all obese patients, and there was no difference among the groups (*P* > 0.05). Compared with the healthy controls, obese patients exhibited greater LV volume (LVEDV, LVESV), LV mass, LV mass index, and LVMMT (all *P* < 0.05). There was no difference in LV concentricity among the groups (*P* > 0.05). For LV strain, the obese group showed lower global LV longitudinal, circumferential, and radial PS and longitudinal and circumferential PDSR than controls (all *P* < 0.05). Among obese patients, the group with hyperlipidemia had lower global longitudinal PS and PDSR and circumferential PDSR than the patients without hyperlipidemia (all *P* < 0.05, Fig. 2). There was no difference in PSSRs in three directions among the groups (*P* > 0.05) (Table 2).

**Table 2:** Comparison of Cardiac Magnetic Resonance Parameters among the four Groups

Parameters	Controls (n = 43)	All obesity (n = 49)	Obesity	
			Hyperlipidemia-(n = 25)	Hyperlipidemia + (n = 24)
LV global function				
LVEF	60.6 ± 5.0	62.7 ± 4.6	62.8 ± 4.6	62.7 ± 4.7
LVEDV (ml)	128 ± 20	158 ± 28*	163 ± 26*	153 ± 30*
LVESV (ml)	51 ± 10	59 ± 13*	61 ± 12*	58 ± 15*
LV mass (g)	75 ± 17	91 ± 20*	91 ± 20*	91 ± 21*
LV mass index (g/m^2.7^)	19.0 ± 3.7	22.3 ± 3.5*	22.5 ± 3.9*	22.1 ± 3.1*
LV concentricity (g/ml)	0.59 ± 0.08	0.57 ± 0.09	0.55 ± 0.08	0.6 ± 0.1
LVMMT (mm)	7.4 ± 0.9	8.0 ± 1.3*	7.7 ± 1.0	8.4 ± 1.5*§
PS (%)				
Longitudinal	− 15.8 ± 2.0	− 13.5 ± 2.9*	− 14.2 ± 2.7*	− 12.8 ± 2.9*§
Circumferential	− 20.6 ± 2.0	− 19.6 ± 2.0*	− 20.0 ± 2.0	− 19.2 ± 2.0*
Radial	35.2 ± 5.4	32.1 ± 5.5*	32.4 ± 5.9*	31.9 ± 5.0*
PSSR (1/s)				
Longitudinal	− 0.8 ± 0.2	− 0.8 ± 0.3	− 0.8 ± 0.2	− 0.8 ± 0.2
Circumferential	− 1.1 ± 0.2	− 1.0 ± 0.3	− 1.0 ± 0.1	− 1.0 ± 0.1
Radial	2.0 ± 0.5	1.9 ± 0.4	1.8 ± 0.3	1.9 ± 0.5
PDSR (1/s)				
Longitudinal	1.1 ± 0.2	0.8 ± 0.2*	0.9 ± 0.3*	0.8 ± 0.1*§
Circumferential	1.5 ± 0.3	1.3 ± 0.2*	1.4 ± 0.2	1.2 ± 0.2*§
Radial	− 2.8 ± 0.7	− 2.6 ± 0.6	− 2.7 ± 0.6	− 2.5 ± 0.5*

Note: *LV* left ventricular; *EF* ejection fraction; *ESV* end-systolic volume; *EDV* end-diastolic volume; *MMT* maximum myocardial thickness; *PS* peak strain; *PSSR* peak systolic strain rate; *PDSR* peak diastolic strain rate *P < 0.05 obese patients versus normal group; §*P* < 0.05 obese patients with hyperlipidemia + versus obese patients with hyperlipidemia -

### Association between hyperlipidemia and LV strain in obesity

Stepwise multivariable linear regression demonstrated that after adjusting for sex, age, height, MAP, FBG, and HOMA-IR, hyperlipidemia was independently associated with longitudinal and circumferential PDSR (β = − 0.362 and β = − 0.477, all *P* < 0.001). Furthermore, after additional adjustment for central fat distribution (visceral fat or android fat or trunk fat), the relationship between LV circumferential PDSR and hyperlipidemia was persisted (β = − 0.477, *P* < 0.001). (Table 3).

**Table 3:** Multivariable Linear Regression Analysis of Association between LV Strains and Hyperlipidemia in Patients with Obesity

	Longitudinal PDSR		Circumferential PDSR	
	Uni-r	Multi-β(R^2^ = 0.299)	Uni-r	Multi-β(R^2^ = 0.228)
Hyperlipidemia	− 0.387*	NS	− 0.477*	− 0.477^§^/− 0.477^§^/− 0.477^§^
Sex	− 0.410*	NS	0.012	NS
Age	0.120	NS	− 0.197*	NS
Height	− 0.217*	NS	0.024	NS
MAP	− 0.258*	NS	− 0.091	NS
FBG	− 0.033	NS	− 0.139	NS
HOMA-IR	− 0.056	NS	− 0.217*	NS
Visceral fat/Android fat/Trunk fat	− 0.518*/− 0.459*/− 0.489*	− 0.518^§^/− 0.459^§^/− 0.489^§^	− 0.370*/− 0.341*/− 0.418*	NS

*BMI* body mass index; *MAP* mean artery pressure; *FBG* fasting blood glucose; *HOMA-IR* homeostasis model assessment of insulin resistance; *PDSR* peak diastolic strain rate

^*^ *P* < 0.1 and ^**§**^ *P* < 0.05

### Association between LV functional parameters and body fat distribution in whole study population

The correlation analysis showed the relationships between LV functional parameters and body fat distribution as follows: (1) DXA-related central fat distribution indices (trunk fat, android fat, and visceral fat) had negative relationships with global PS and PDSR in longitudinal, circumferential, and radial directions (r = − 0.252 to − 0.563); (2) DXA-related peripheral fat distribution indices (peripheral fat and gynoid fat) had positive relationships with global PS and PDSR in three directions (r = 0.286–0.529); (3) BMI had a negative correlation with global PS, longitudinal PSSR and PDSR, and circumferential PDSR (r = − 0.214 to − 0.446); (4) WC was inversely associated with global longitudinal PS and PDSR and circumferential PS (r = − 0.222 to − 0.383); (5) waist-to-hip ratio was negatively associated with global longitudinal PS and PDSR (r = − 0.315 and − 0.360); (6) EAT was negatively associated with global longitudinal PS and PDSR and circumferential PDSR (r = − 0.283 to − 0.352) (Table 4).

**Table 4:** Correlation Coefficients between Left Ventricular Strain and Body Fat Distribution in Whole Population

	BMI	WC	Waist-to-hip ratio	Trunk fat	Peripheral fat	Android fat	Gynoid fat	Visceral fat	EAT
PS (%)									
Longitudinal	− 0.446^**^	− 0.383^**^	− 0.315^**^	− 0.493^**^	0.459^**^	− 0.525^**^	0.410^**^	− 0.516^**^	− 0.323^**^
Circumferential	− 0.226^*^	− 0.222^*^	− 0.174	− 0.284^**^	0.318^**^	− 0.273^**^	0.393^**^	− 0.291^**^	− 0.177
Radial	− 0.273^**^	− 0.205	− 0.143	− 0.305^**^	0.286^**^	− 0.316^**^	0.304^**^	− 0.252^*^	− 0.151
PSSR (1/s)									
Longitudinal	− 0.214^*^	− 0.121	− 0.087	− 0.152	0.099	− 0.211^*^	0.108	− 0.201	− 0.105
Circumferential	− 0.162	− 0.056	− 0.038	− 0.111	0.050	− 0.144	0.078	− 0.114	− 0.111
Radial	− 0.183	− 0.040	0.008	− 0.145	0.103	− 0.168	0.094	− 0.131	− 0.081
PDSR (1/s)									
Longitudinal	− 0.429^**^	− 0.374^**^	− 0.360^**^	− 0.505^**^	0.529^**^	− 0.526^**^	0.497^**^	− 0.563^**^	− 0.352^**^
Circumferential	− 0.284^**^	− 0.194	− 0.171	− 0.368^**^	0.408^**^	− 0.356^**^	0.481^**^	− 0.424^**^	− 0.283^**^
Radial	− 0.137	− 0.135	− 0.117	− 0.262^*^	0.325^**^	− 0.297^**^	0.413^**^	− 0.362^**^	− 0.163

Note: All strain parameters are calculated as absolute values. *PS* peak strain; *PSSR* peak systolic strain rate; *PDSR* peak diastolic strain rate; *BMI* body mass index; *WC* waist circumference; *EAT* epicardial adipose tissue

^*^ *P* < 0.05 and ^**^ *P* < 0.01

**Fig. 3:**
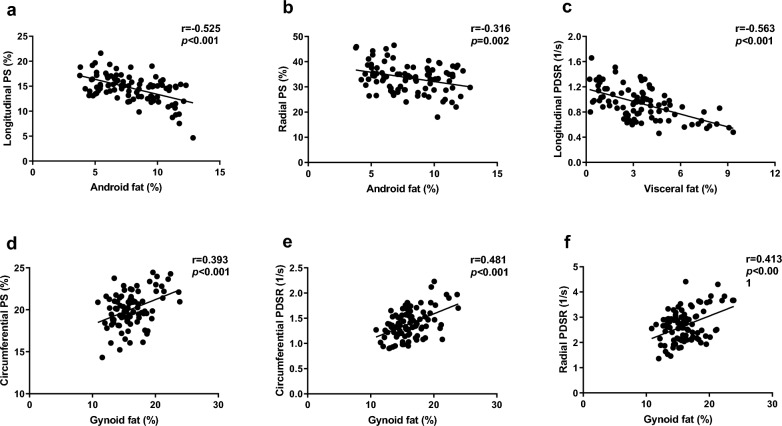
Correlations between regional fat distribution and left ventricular strain parameters. **a** and **b** show inverse correlations between android fat with longitudinal and radial PS; **c** demonstrates a negative correlation between visceral fat and longitudinal PDSR. **d**, **e** and **f** show that gynoid fat is positively associated with circumferential PS, circumferential PDSR, and radial PDSR. *PS* peak strain, *PDSR* peak diastolic strain rate

After adjusting for sex, age, height, and related body fat distribution parameters with a *P* < 0.1 in the univariable analyses, a stepwise multivariable linear regression demonstrated that LV longitudinal and radial PS had independently negative relationships with android fat (β = − 0.486, β = − 0.408; all *P* < 0.001); LV longitudinal PDSR was independently correlated with visceral fat (β = − 0.563, *P* < 0.001); conversely, LV circumferential PS and PDSR and radial PDSR were positively correlated with gynoid fat (β = 0.490, β = 0.481, β = 0.413, respectively; all *P* < 0.001). (Table 5 and Fig. 3).

**Table 5:** Multivariable Linear Regression Analysis of Association between Left Ventricular Strain Parameters and Body Fat Distribution in Whole Population

Independent variables	R square	Factors in models	B	β	*P* value
Longitudinal PS	0.312	Android fat%	− 0.556	− 0.486	< 0.001
		Sex	− 1.065	− 0.194	0.034
Circumferential PS	0.213	Gynoid fat%	0.372	0.490	< 0.001
		age	0.065	0.261	0.012
Radial PS	0.179	Android fat%	− 0.915	− 0.408	< 0.001
		age	0.202	0.296	0.004
Longitudinal PDSR	0.317	Visceral fat%	− 0.067	− 0.563	< 0.001
Circumferential PDSR	0.231	Gynoid fat%	0.050	0.481	< 0.001
Radial PDSR	0.170	Gynoid fat%	0.098	0.413	< 0.001

Note: All strain parameters are calculated as absolute values. *PS* peak strain; *PDSR* peak diastolic strain rate

### Association between body fat distribution and cardiovascular risk factors in whole study population

After controlling for age and sex, android fat, visceral fat, and trunk fat were positively correlated with triglycerides, total cholesterol, LDL, VLDL, FBG, FINS, HOMA-IR, and MAP (r = 0.270–0.648), while negatively correlated with HDL (r = − 0.549 to − 0.595). In contrast, peripheral fat and gynoid fat had negative associations with triglycerides, total cholesterol, LDL, VLDL, FBG, HOMA-IR, and MAP (r = − 0.226 to − 0.593), whereas positive associations with HDL (r = 0.524 and r = 0.436, respectively) (Table 6).

**Table 6:** Association between Body Fat Distribution and Cardiovascular Risk Factors after Controlling for Age and Sex in Whole Population

	Triglycerides	Total cholesterol	HDL	LDL	VLDL	FBG	FINS	HOMA-IR	MAP
Android fat%	0.610^*^	0.369^*^	− 0.595^*^	0.329^*^	0.609^*^	0.432^*^	0.518^*^	0.627^*^	0.482^*^
Gynoid fat%	− 0.459^*^	− 0.277^*^	0.436^*^	− 0.226^*^	− 0.459^*^	− 0.250^*^	− 0.282^*^	− 0.521^*^	− 0.319^*^
Visceral fat%	0.613^*^	0.361^*^	− 0.549^*^	0.270^*^	0.612^*^	0.348^*^	0.335^*^	0.535^*^	0.436^*^
Trunk fat%	0.599^*^	0.354^*^	− 0.587^*^	0.301^*^	0.599^*^	0.373^*^	0.376^*^	0.648^*^	0.518^*^
Peripheral fat%	− 0.558^*^	− 0.354^*^	0.524^*^	− 0.298^*^	− 0.558^*^	− 0.298^*^	− 0.527^*^	− 0.593^*^	− 0.418^*^

Note: *HDL* high-density lipoprotein; *LDL* low-density lipoprotein; *VLDL* very-low-density lipoprotein; *FBG* fasting blood glucose; *FINS* fasting insulin; *HOMA-IR* homeostasis model assessment of insulin resistance; *MAP* mean artery pressure; *P < 0.05

### Intra- and interobserver variability

Good intra- and interobserver agreement was observed for LV global function and geometry (ICC 0.946–0.982 and 0.930–0.977, respectively), LV global strain (ICC 0.925–0.932 and 0.906–0.921, respectively), and LV global strain rate (PSSR and PDSR) (ICC 0.845–0.902 and 0.832–0.874, respectively). (Additional file 1: Table S1).

## Discussion

In this study, we compared LV geometry and function among obese (hyperlipidemia +), obese (hyperlipidemia-), and healthy control subjects using CMR and assessed the association between LV strain and body fat distribution. The principal findings were as follows: (1) the obese group with preserved LVEF had impaired subclinical LV function manifesting as diminished radial, longitudinal, and circumferential PS and longitudinal and circumferential PDSR compared with healthy controls; (2) among obese patients, those with hyperlipidemia had lower global longitudinal PS and PDSR and circumferential PDSR than those without hyperlipidemia; (3) the hyperlipidemia was independently associated with subclinical LV diastolic dysfunction; (4) Central fat distribution (android fat and visceral fat) has a negative and peripheral fat distribution (gynoid fat) a positive impact on subclinical LV function.

### Hyperlipidemia may aggravate subclinical LV dysfunction in obesity

Our study showed that although all LVEFs were within the normal range in both obese and healthy individuals, obese subjects had impaired radial, longitudinal, and circumferential PS and longitudinal and circumferential PDSR compared with healthy controls. The results are consistent with previous studies on CMR in adults and children without hypertension, diabetes, and heart disease, indicating that these obese patients had subclinical LV dysfunction with preserved LVEF [22–24]. This finding was explained by capacity overload, increased blood pressure, LV structural remodelling (increased myocardial mass, wall thickness, and chamber size), insulin resistance, and increased EAT volume in obesity in our previous study [22]. Furthermore, although the obese individuals did not have hypertension, diabetes, and cardiovascular disease, they had elevated triglycerides, total cholesterol, VLDL, and LDL and reduced HDL levels compared with the healthy controls. To determine the association of hyperlipidemia with LV function in obese patients, we divided them into two subgroups based on the presence of hyperlipidemia. We found that those obese patients with hyperlipidemia had lower global longitudinal PS and PDSR and circumferential PDSR than those without hyperlipidemia. In addition, hyperlipidemia was independently associated with subclinical LV diastolic dysfunction in obesity even after controlling for growth differences, other metabolic-related cardiovascular risk factors, and central fat distribution. Previous echocardiographic studies also indicated that metabolically unhealthy obese patients (i.e., those diagnosed with metabolic syndrome) had lower subclinical LV diastolic and/or systolic function than obese patients who were metabolically healthy [25, 26]. Compared with past studies, ours excluded obese individuals with diabetes and hypertension and focused on the specific effect of hyperlipidemia on subclinical LV function in obesity.

Elevated free fatty acid (FFA) levels promote VLDL synthesis in the liver, hypertriglyceridemia, reduced HDL concentration, and the formation of small, dense LDL particles, raising the risk of cardiovascular disease [27]. A previous study also showed that obese women had higher glycerol and FFA rates than normal subjects [28]. In addition, Peterson et al. [29] showed that obese women have increased myocardial FFA uptake and oxygen consumption. Despite an increase in myocardial FFA oxidation in obesity, there is evidence of lipid accumulation in human hearts using either magnetic resonance spectroscopy or lipid staining of postmortem tissue [30, 31], indicating an imbalance in myocardial fatty acid metabolism, with absorption surpassing oxidation. Thus, hyperlipidemia may be considered as a marker of increased fatty acids and myocardial lipid deposition. Evidences from animal studies also indicated that excessive accumulation and oxidation of lipids in the myocardium results in cardiomyocyte apoptosis and myocardial dysfunction, in which ceramide, oxidative stress, and reactive oxygen species production may be intermediate steps [32–35].

Recent studies also showed that dyslipidemia was related to higher levels of inflammatory markers, such as interleukin-6 (IL-6), tumor necrosis factor alpha-a (TNF-α), and C-reactive protein (CRP) [36–38]. IL-6 played a crucial role in aldosterone-induced macrophage recruitment and infiltration of myocardial macrophages, causing myocardial fibrosis [39]. TNF-α activates sphingomyelinase to catalyze the sphingomyelin hydrolysis to ceramide [40]. CRP and TNF-α have been associated with insulin resistance and atherosclerosis. Earlier studies demonstrated that myocardial fibrosis, ceramide accumulation, and insulin resistance were associated with cardiomyocyte apoptosis and LV dysfunction [22, 32]. In addition, Mahemuti et al. reported the link between hyperlipidemia and systemic immunity-inflammation index, a novel inflammatory marker [41]. A recent study with a large sample size demonstrated a strong association between elevated systemic immunity-inflammation index levels and heart failure [42]. Increased inflammation may be a potential mechanism to explain how hyperlipidemia was associated with impaired LV function. More researches are needed to elucidate this effect.

In addition, our study found no significant association between FBG and LV strain in obese patients. Prior studies showed that hyperglycemia was associated with impaired LV strain in patients with diabetes mellitus, whereas there was no significant difference in LV strain in patients with impaired glucose tolerance alone compared with healthy controls [43, 44]. Our study excluded people with diabetes mellitus and most had normal fasting glucose, which may explain the lack of significant correlation between FBG and LV strain. In addition, a mendelian randomization study also indicated that glycemic levels were no associated with LV structure and function [45].

### Body fat distribution and subclinical LV function

Our study assessed the relationship between regional fat distribution and LV function in whole study population. Our result demonstrated that central fat distribution (visceral and android fat) had a negative association, while peripheral fat distribution (gynoid fat) had a positive association on LV function. Prior study have also revealed that VAT is independently associated with impairment of LV strain, while lower-body fat is not associated with it [17].

One explanation for the inconsistency may be related to the different effects of body fat distribution on metabolic disorders. Our findings showed that after controlling for age and sex, visceral and android fat were positively associated with triglycerides, total cholesterol, LDL, FBG, MAP, and HOMA-IR, but negatively correlated with HDL. In contrast, peripheral fat distribution had the opposite effect on these parameters. Previous researches also suggested that android and visceral fat had a positive association with cardiometabolic risk factors (hypertriglyceridemia, impaired fasting glucose, elevated blood pressure, and insulin resistance), whereas gynoid fat had a negative association with these factors [18, 46–48]. As discovered in this study, hyperlipidemia impaired LV function in obese patients. Moreover, insulin resistance was independently associated with subclinical LV dysfunction (shown as decreased circumferential PS and PDSR) in obese patients without hypertension, diabetes, and other cardiovascular diseases [22]. In addition, central fat distribution increased the risk of hypertension, while peripheral fat distribution decreased this risk [46]. Lastly, hypertension was a significant risk factor for LV diastolic dysfunction [49].

Another explanation may be chronic inflammatory factors and adipokines related to obesity. Visceral and android fat were inversely associated with adiponectin [50], while positively associated with leptin [51]. In addition, lower extremity fat was positively associated with adiponectin [52]. Adiponectin as a cardioprotective adipokine mediated insulin-sensitizing effects and reduces hyperlipidemia [53]. Leptin plays a beneficial role in enhanced insulin sensitivity and the inhibition of food intake. However, hyperleptinemia in obesity principally resulted from leptin resistance, which had a pro-inflammatory role [54]. Abdominal fat and visceral fat had positive correlations with TNF-α and CRP; in contrast, peripheral fat (lower extremity fat, thigh subcutaneous fat, or gynoid fat) had an inverse correlation with these factors [47, 50, 52]. As mentioned above, CRP and TNF-α were associated with insulin resistance and ceramide production, which may be associated with reduced LV function.

In summary, the association between central or peripheral adiposity and LV strain may be partially mediated through its effects on metabolic related cardiovascular risk factors, systemic inflammation, or adipokines. Our study suggested the importance of categorizing obese subjects into different types of obesity when investigating its associations on LV function.

There were several limitations in this study. First, it was cross-sectional. We cannot determine causal associations of hyperlipemia and regional fat distribution with LV strain. Second, this was an exploratory study with limited sample size, especially in the subgroup of obese patients (hyperlipidemia- and hyperlipidemia +). Therefore, further confirmation of our findings is required in large sample studies. Third, although previous studies have reported intramyocardial triglycerides accumulation (myocardial lipotoxicity) in obesity, myocardial lipid accumulation was not quantified in our study. Finally, our study reported the relationships between regional fat distributions and metabolic related cardiovascular risk factors to explain the effect of regional fat distributions on LV function. However, more data, such as inflammatory markers and cytokines are needed to understand the different associations of regional fat distributions with LV function.

## Conclusion

In obesity, hyperlipidemia is independently associated with subclinical LV diastolic dysfunction assessed by strain analysis. Central fat distribution (android and visceral fat) has a negative association, while peripheral fat distribution (gynoid fat) has a positive association on subclinical LV function. The results might help determine appropriate strategies for the management of patients with obesity.

### Supplementary Information

**Additional file 1: Table S1.** Comparison of Inter- and Intra-Observer Variability of CMR Measures.

## Data Availability

The datasets generated and analyzed during the current study are available from the corresponding authors on reasonable request.

## Abbreviations

BMI: Body mass index
CMR: Cardiovascular magnetic resonance
CRP: C-reactive protein
DBP: Diastolic blood pressure
EAT: Epicardial adipose tissue
EDV: End diastolic volume
EF: Ejection fraction
ESV: End systolic volume
FBG: Fasting blood glucose
FINS: Fasting insulin
HC: Hip circumference
HDL: High-density lipoprotein
HOMA-IR: Homeostasis model assessment of insulin resistance
IL-6: Interleukin-6
LDL: Low-density lipoprotein
LV: Left ventricular
MAP: Mean artery pressure
MMT: Maximum myocardial thickness
PDSR: Peak diastolic strain rate
PS: Peak strain
PSSR: Peak systolic strain rate
SBP: Systolic blood pressure
TNF-α: Tumor necrosis factor alpha-a
VLDL: Very-low-density lipoprotein
WC: Waist circumference
