# Supplementary material for: The effect of hyperlipidemia and body fat distribution on subclinical left ventricular function in obesity: a cardiovascular magnetic resonance study
Source: Cardiovasc Diabetol. 2024 Apr 2;23:120. doi: 10.1186/s12933-024-02208-z (PMC10985902; doi:10.1186/s12933-024-02208-z)
Supplement: Supplementary file 1 — Additional file 1: Table S1. Comparison of Inter- and Intra-Observer Variability of CMR Measures. [file 12933_2024_2208_MOESM1_ESM.docx]

| **Table S1: Comparison of Inter- and Intra-Observer Variability of CMR Measures.** | | | | | |
| --- | --- | --- | --- | --- | --- |
|  | Intra-observer (n = 20) | |  | Inter-observer (n = 20) | |
|  | ICC | 95% CI |  | ICC | 95% CI |
| **LV global function and geometry** |  |  |  |  |  |
| LVEF | 0.982 | 0.966 – 0.993 |  | 0.972 | 0.944 – 0.988 |
| LVEDV | 0.980 | 0.959 – 0.989 |  | 0.977 | 0.950 – 0.988 |
| LVESV | 0.974 | 0.945 – 0.990 |  | 0.970 | 0.945 – 0.986 |
| LV mass | 0.973 | 0.944 – 0.988 |  | 0.969 | 0.936 – 0.989 |
| LVMMT | 0.946 | 0.918 – 0.967 |  | 0.930 | 0.889 – 0.956 |
| **PS (%)** |  |  |  |  |  |
| Radial | 0.928 | 0.812-0.991 |  | 0.914 | 0.829 - 0.945 |
| Circumferential | 0.932 | 0.842-0.989 |  | 0.921 | 0.827-0.950 |
| Longitudinal | 0.925 | 0.844-0.981 |  | 0.906 | 0.841 - 0.931 |
| **PSSR (s^-1^)** |  |  |  |  |  |
| Radial | 0.880 | 0.736- 0.956 |  | 0.874 | 0.712 - 0.951 |
| Circumferential | 0.902 | 0.799 - 0.940 |  | 0.840 | 0.700 - 0.929 |
| Longitudinal | 0.845 | 0.734 - 0.903 |  | 0.832 | 0.710 - 0.900 |
| **PDSR (s^-1^)** |  |  |  |  |  |
| Radial | 0.882 | 0.740 - 0.956 |  | 0.860 | 0.705 - 0.950 |
| Circumferential | 0.854 | 0.732 - 0.936 |  | 0.851 | 0.730 - 0.930 |
| Longitudinal | 0.868 | 0.762 - 0.938 |  | 0.853 | 0.755 - 0.931 |
| LV=left ventricular; EF = ejection fraction; ESV = end-systolic volume; EDV = end-diastolic volume; MMT = maximum myocardial thickness; PS=peak strain, PDSR=peak diastolic strain rate, PSSR=peak systolic strain rate | | | | | |
